# Comparison of inflammatory markers and microflora in cirrhotic patients with portal vein thrombosis: a retrospective study

**DOI:** 10.3389/fmed.2025.1680970

**Published:** 2026-01-12

**Authors:** Yanzhao Sun, Cunkai Wang, Dingxin Wang, Hongtao Hou, Jian Zhang, Yueqin Li, Yun Bai

**Affiliations:** Department of Geriatric Gastroenterology, Hebei General Hospital, Shijiazhuang, China

**Keywords:** hepatic cirrhosis, inflammatory markers, microbiota, oxidative stress, portal vein thrombosis

## Abstract

**Background:**

This study investigates inflammatory markers and blood microflora in cirrhotic patients with portal vein thrombosis (PVT).

**Aims:**

This study explores inflammatory markers and microflora in cirrhotic patients with PVT.

**Methods:**

This retrospective study at Hebei General Hospital between September 2021 and December 2023 categorized patients into PVT and non-PVT groups. Portal and peripheral vein blood samples were collected during TIPS procedure, with inflammatory markers LPS, IL-6, IL-8, TNFα and sNox2-dp tested via ELISA and 16SrRNA sequencing of blood microflora.

**Results:**

The study included 28 cirrhotic patients: 12 in PVT group (5 males, mean age: 55.8 ± 11.2) and 16 in non-PVT group (13 males, mean age: 54.3 ± 13.6). Groups showed no differences in age, gender, or BMI(*P* > 0.05), but D-dimer levels were higher in PVT group (*P* < 0.05). Portal vein blood in PVT patients showed elevated LPS (34.42 vs. 13.59 pg/mL), IL-6 (38.46 vs. 18.76 pg/mL), TNFα (46.74 vs. 18.92 pg/mL), and sNox2-dp (13.03 vs. 5.13 pg/mL) vs. non-PVT patients (*P* < 0.05), with no peripheral blood differences.

**Conclusion:**

Pseudomonas and Microbacteriaceae were enriched in PVT portal blood. Rolstonia, Clostridium, Phenylobacterium, and Streptococcus correlated with LPS and IL-6 (*P* < 0.05), while Phenylobacterium linked to D-dimer (*P* < 0.05). Cirrhotic patients with PVT show higher portal vein inflammatory markers due to bacterial translocation.

## Introduction

Portal vein thrombosis (PVT) is defined as thrombosis of the main portal vein and/or the left and right branches of the intrahepatic portal vein, with or without mesenteric vein and splenic vein thrombosis (1). PVT is one of the most common complications in cirrhotic patients in the advanced stages of the disease, with 10% in compensated cirrhosis (2), 5% to 18% in decompensated cirrhosis (3), and 26% in patients undergoing liver transplantation (4). PVT is associated with the severity of liver injury, with its prevalence and cumulative incidence associated with the loss of liver functional reserve and decline of liver function, which further affects the prognosis of cirrhotic patients. The underlying mechanism of PVT formation is multifaceted (5). The widely recognized Virchow's Theory (including reduced flow, hypercoagulability, and vascular endothelial injury) of venous thrombosis also applies to PVT, specifically, Kinjo et al. (6) showed portal venous flow was greatly reduced in the PVT patients, Xing et al. (7) showed that hypercoagulability markers of cirrhosis patients with PVT were significantly higher than those of cirrhosis patients without PVT, and Guo et al. (8) confirmed the role of vascular endothelial injury in the development of PVT using rat model.

However, apart from the aforementioned factors, PVT may be associated with bacterial translocation, endotoxemia, and systemic inflammation. Lipopolysaccharide (LPS) has been identified as a key factor (9) that plays a crucial role in promoting the release of inflammatory mediators, such as IL-6: interleukin-6. IL-8: interleukin-8, tumor necrosis factor α (TNFα) in vein blood. Additionally, it enhances platelet adhesion, aggregation, activation, and thrombosis by increasing oxidative stress (sNox2-dp). Meanwhile, other studies have reported that bacterial translocation and bacterial products (9) (e.g., endotoxin) can lead to hepatic encephalopathy and spontaneous bacterial peritonitis, which could also increase inflammatory markers. Theoretically, although the mechanism underlying PVT and microflora change was not fully understood, the association between PVT and gut dysbiosis was confirmed in previous studies, after microbiota dysbiosis-induced gut barrier dysfunction, lipopolysaccharides may translocate into systemic circulation, while the relation between PVT and blood microflora dysbiosis has not been less studied (10). Given these complex and interrelated mechanisms underlying PVT development, a more comprehensive understanding of the interplay between various factors is essential. Therefore, this study aims to explore the inflammatory markers and microflora in cirrhotic patients with PVT.

## Methods

### Study design and population

This retrospective study enrolled patients with hepatitis B cirrhosis at Hebei General Hospital between September 2021 and December 2023. The inclusion criteria were: (1) patients diagnosed with Cirrhosis; (2) For patients with insufficient evidence of cirrhosis but imaging findings suggestive of PVT, further diagnosis of cirrhosis was confirmed via hepatic venous pressure gradient (HVPG) measurement and transjugular liver biopsy; (3) patients with complete laboratory examination and 16SrRNA sequencing results; (4) patients aged 30–78 years. The exclusion criteria were: (1) Patients with primary or secondary hepatic malignancies; (2) Patients with other concurrent malignancies; (3) Patients with hematologic disorders; (4) Patients with Budd-Chiari syndrome or PVT caused by non-cirrhotic etiologies; (5) Patients with severe infections; (6) Patients requiring anticoagulant therapy for major conditions such as myocardial infarction, cerebral infarction, or pulmonary embolism; (7) Patients with hepatic encephalopathy or liver failure. The study was approved by the ethical review committee of Hebei General Hospital (NO.2022140). The requirement for individual consent was waived by the committee because of the retrospective nature of the study.

### Treatment procedure

####  TIPS

The TIPS was performed in two groups of patients: (1) Patients with Child-Pugh class B and endoscopically confirmed active bleeding; and (2) Patients with Child-Pugh class C and a score ≤ 13. The vascular access was achieved via the right internal jugular vein. Following catheterization of the superior vena cava, the procedure was performed with stent placement targeting the left branch of the portal vein.

#### Medication

Rifaximin and probiotics were not administered in any patient, while antibiotics (specifically, third-generation cephalosporins) were indicated in patients with cirrhosis and bleeding.

### Data collection and definition

Basic information (including age, gender) and laboratory examination results (including platelet count (PLT), hemoglobin (Hb), white blood cell count (WBC), D-dimer, prothrombin time (PT), international normalized ratio (INR), albumin (Alb), total bilirubin, creatinine, Child-pugh score, MELD scores, LPS), IL-6, IL-8, TNFα, and sNox2-dp) of patients were collected from medical records. The levels of LPS, IL-6, IL-8, TNFα, and sNox2-dp were measured using enzyme-linked immunosorbent assay (Jiangsu Jingmei Biotechnology Co., Ltd.). And the 16SrRNA sequencing was obtained (Shanghai Parasen Pharmaceutical Biotechnology Co., Ltd.). The total DNA of frozen blood samples was extracted with the Blood Genomic DNA Extraction Kit (Shanghai Parasen Pharmaceutical Biotechnology Co., Ltd.). The concentration and purity of the samples were detected with a UV spectrophotometer after volume fixation to 50 μL, followed by PCR amplification, Miseq library construction, and Miseq sequencing. The portal hypertension was also assessed in all patients. The inclusion criteria mandated a baseline hepatic venous pressure gradient (HVPG) of at least 12 mmHg.

### Statistical analysis

Statistical analyses were performed using SPSS 26.0 (IBM Corp., New York, USA) and GraphPad Prism 9 (GraphPad Software, San Diego, USA). Categorical data were expressed as frequencies and percentages, using χ^2^ or Fisher's exact test. Continuous data that conformed to normal distribution were expressed as mean ± standard deviation (SD), using a *t*-test, while continuous data that were not normally distributed were expressed as median and interquartile range. The colony α-diversity assessed by chao1 and shannon indices, while colony β-diversity was compared by principal coordinate analysis (PCoa) plots, species with significant differences in abundance between specified taxa identified by linear discriminant analysis (lefse), the effect of how each component (species) abundance affected on differential effects estimated by linear regression analysis (LDA). Two-sided *P* < 0.05 was defined as statistically significant.

## Results

### Basic characteristics

Twenty-eight cirrhotic patients who underwent TIPS were included for final analysis; among them, 12 patients with PVT were classified into the PVT group, while 16 were classified into the non-PVT group. Compared to patients in the non-PVT group, patients in the PVT showed significant higher D-dimer [3.50 (1.37–12.81) vs. 1.46 (0.83–3.37), *P* = 0.028], while for other basic characteristics and laboratory examination results, there was no significant difference in age, gender distribution, BMI, PLT, Alb, total bilirubin, creatinine, PT, PTA, FIB, INR, Child-push score, Child-push grading and MELD score between both groups (all *P* > 0.05) (Table 1).

**Table 1:** Basic characteristics.

**Variables**	**PVT**	**Non-PVT**	***P***
Gender (n[%])			0.065
Male	5 (41)	13 (76)	
Female	7 (59)	4 (24)	
Age (years)	55.8 ± 11.2	54.3 ± 13.6	0.704
BMI (kg/m^2^)	23.4 ± 2.2	25.7 ± 3.8	0.386
PLT (× 10^∧^9/L)	61.08 ± 26.02	74 ± 40.47	0.306
Alb (g/L)	32.1 ± 2.9	31.5 ± 5.06	0.688
Total bilirubin (μmol/L)	21.8 (15.1–30.5)	19.5 (12.7–50.1)	0.945
Creatinine (μmol/L)	70.4 ± 14.2	78.8 ± 13.7	0.145
PT (S)	12.75 (12.1–13.8)	13.9 (13.5–17.4)	0.051
FIB (g/L)	2.26 (1.83–3.90)	2.07 (1.66–2.29)	0.146
D-dimer (mg/L)	3.50 (1.37–12.81)	1.46 (0.83–3.37)	**0.028**
INR	1.18 (1.01–1.45)	1.22 (1.18–1.52)	0.241
Child-pugh score	8 (7.25–9)	8 (7–9)	0.303
Child-pugh grading			0.556
A	0 (0)	0 (0)	
B	10 (83)	15 (88)	
C	2 (17)	2 (12)	
MELD score	5.00 (4.08–9.09)	7.5 (6.48–11.52)	0.079

BMI, Body Mass Index; PLT, Platelet Count; Alb, Albumin; PT, Prothrombin Time; FIB, Fibrinogen; INR, International Normalized Ratio. A statistically significant difference in D-dimer levels was observed between the PVT and non-PVT groups.

### Comparison of inflammatory markers between PVT and non-PVT groups

**Figure 1:**
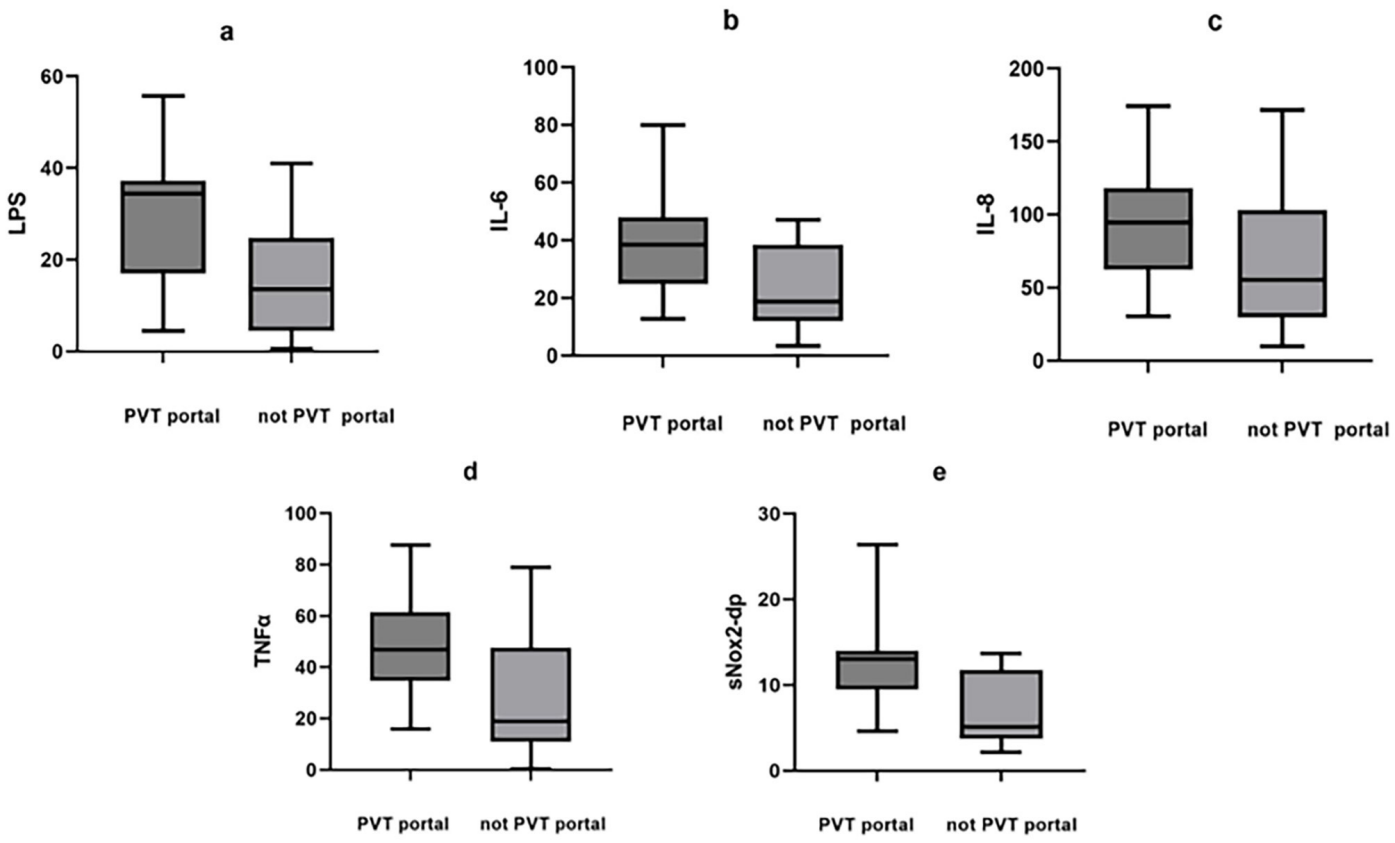
Inflammatory markers in portal blood between PVT and non-PVT groups. **(a)** LPS, **(b)** IL-6, **(c)** IL-8, **(d)** TNFα, **(e)** sNox2-dp.

**Figure 2:**
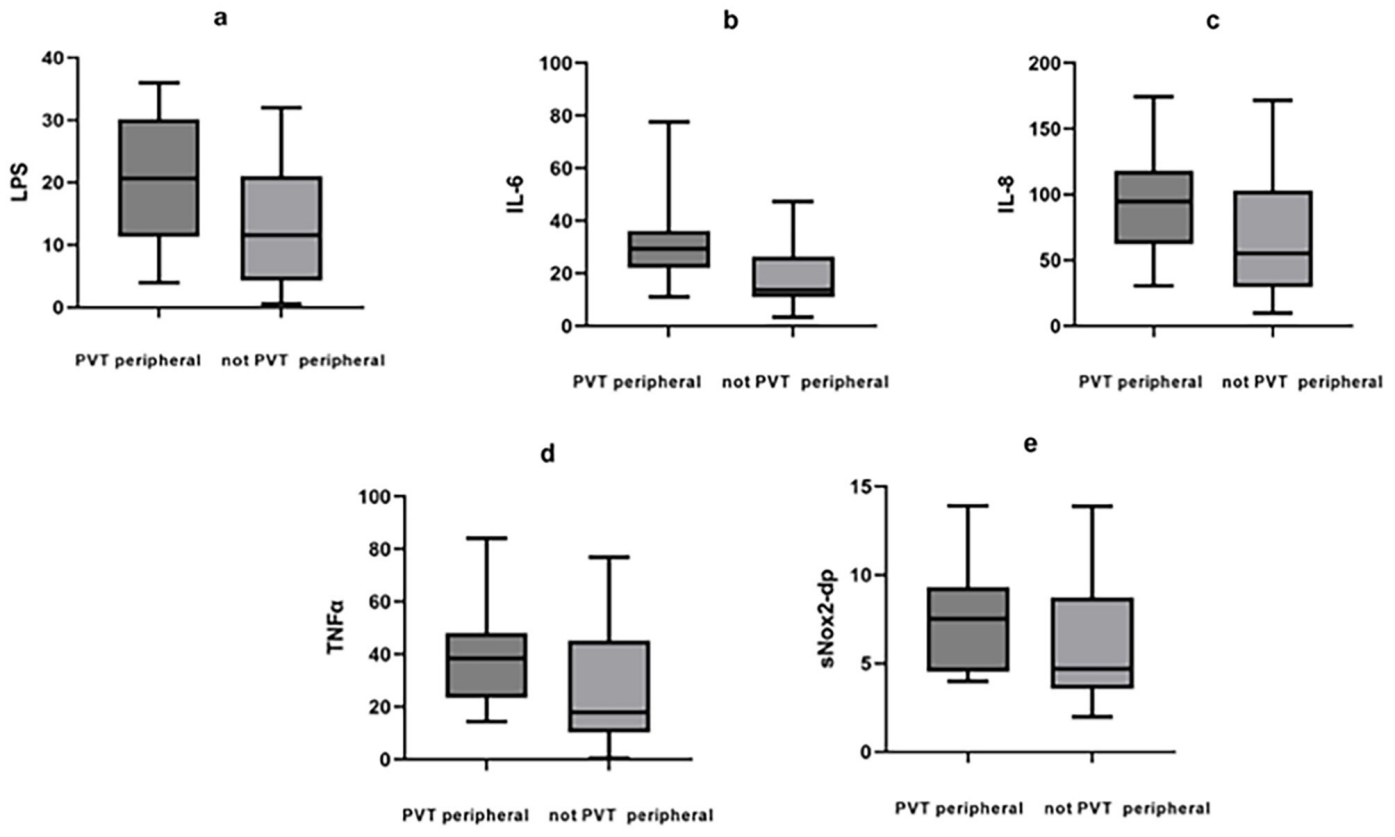
Inflammatory markers in peripheral venous blood between PVT and non-PVT groups. **(a)** LPS, **(b)** IL-6, **(c)** IL-8, **(d)** TNFα, **(e)** sNox2-dp.

The concentration of inflammatory markers of portal vein blood was also compared in PVT and non-PVT groups, and the results showed that LPS (34.42 vs. 13.59 pg/mL, *P* < 0.05), IL-6 (38.46 vs. 18.76 pg/mL, *P* < 0.05), TNFα (46.74 vs. 18.92 pg/mL, *P* < 0.05), and sNox2-dp (13.03 vs. 5.13 pg/mL, *P* < 0.05) were all significantly higher in portal vein blood obtained from patients of PVT group (Table 2; Figure 1). While there was no statistically significant difference in the above inflammatory markers in peripheral vein blood, with the inflammatory levels as LPS (20.61 vs. 11.55 pg/mL, *P* > 0.05), IL-6 (29.35 vs. 13.63 pg/mL, *P* > 0.05), IL-8 (46.74 vs. 50.96 pg/mL, *P* > 0.05), TNFα (38.45 vs. 28.08 pg/mL, *P* > *0.05*), and sNox2-dp (7.50 vs. 4.69 pg/mL, *P* > 0.05) (Table 2; Figure 2).

**Table 2:** Inflammatory markers between PVT and non-PVT groups.

**Variables**	**PVT**	**Non-PVT**	***P***
**Portal vein blood**			
LPS (pg/ml)	34.42 (20.21–39.32)	13.59 (8.56–21.10)	0.011
IL-6 (pg/ml)	38.46 (26.75–50.31)	18.76 (15.52–31.18)	0.037
IL-8 (pg/ml)	94.37 (68.95–117.62)	55.23 (42.96–95.85)	0.104
TNFα (pg/ml)	46.74 (34.98–62.22)	18.92 (17.69–44.5)	0.041
sNox2-dp (pg/ml)	13.03 (8.87–16.02)	5.13 (5.15–9.55)	0.009
**Peripheral vein blood**			
LPS (pg/ml)	20.61 (13.01–26.82)	11.55 (7.76–18.56)	0.104
IL-6 (pg/ml)	29.35 (20.4–42.72)	13.63 (12.63–27.35)	0.063
IL-8 (pg/ml)	46.74 (52.66–101.45)	50.96 (36.06–85.78)	0.104
TNFα (pg/ml)	38.45 (27.06–54.38)	28.08 (15.69–42.60)	0.086
sNox2-dp (pg/ml)	7.50 (5.42–9.34)	4.69 (4.40–8.65)	0.236

LPS, lipopolysaccharide; IL-6, interleukin-6; IL-8, interleukin-8; TNFα, tumor necrosis factor α.

### Comparison of inflammatory markers between portal and peripheral veins blood

The concentration of inflammatory markers were compared between the portal vein blood and peripheral venous blood, the results showed that in 28 cirrhotic patients, the concentration of LPS (23.37 vs. 18.79 pg/mL, *P* < 0.05), IL-6 (29.85 vs. 24.95 pg/mL, *P* < 0.05), IL-8 (79.64 vs. 67.84 pg/mL, *P* < 0.05), TNFα (38.24 vs. 34.46 pg/mL, *P* < 0.05), and sNox2-dp (9.53 vs. 7.89 pg/mL, *P* < 0.05) were all higher in the portal vein blood (Table 3; Supplementary Figure S1). The subgroup analysis showed that in patients of PVT group, the concentration of LPS (29.76 vs. 19.92 pg/mL, *P* < 0.05), IL-6 (38.53 vs. 31.56 pg/mL, *P* < 0.05), IL-8 (93.28 vs. 77.05 pg/mL, *P* < 0.05), TNFα (48.60 vs. 40.55 pg/mL, *P* < 0.05), and sNox2-dp (12.45 vs. 7.38pg/mL, *P*<*0.05*) were also higher in the portal vein blood than in the peripheral vein blood (Table 3; Supplementary Figure S2).

**Table 3:** Inflammatory markers between portal and peripheral venous blood.

**Varibles**	**Portal vein blood**	**Peripheral vein blood**	***P***
**Cirrhotic patients**			
LPS (pg/ml)	23.37 (4.40–37.26)	18.79 (4.40–37.26)	<0.001
IL-6 (pg/ml)	29.85 (12.80–44.01)	24.95 (4.40–37.27)	<0.001
IL-8 (pg/ml)	79.64 (46.18–104.88)	67.84 (4.40–37.28)	<0.001
TNFα (pg/ml)	38.24 (16.79–57.81)	34.46 (4.40–37.29)	0.007
sNox2-dp (pg/ml)	9.53 (4.66–13.60)	7.89 (4.39–9.90)	<0.001
**PVT patients**			
LPS (pg/ml)	29.76 (17.01–37.26)	19.92 (11.33–30.15)	0.002
IL-6 (pg/ml)	38.53 (24.82–48.06)	31.56 (22.11–36.13)	0.003
IL-8 (pg/ml)	93.28 (62.4–118.28)	77.05 (52.41–94.59)	0.005
TNFα (pg/ml)	48.60 (34.59–61.56)	40.55 (23.52–38.03)	0.002
sNox2-dp (pg/ml)	12.45 (9.48–14.01)	7.38 (4.52–9.31)	0.005

LPS, lipopolysaccharide; IL-6, interleukin-6; IL-8, interleukin-8; TNFα, tumor necrosis factor α.

**Figure 3:**
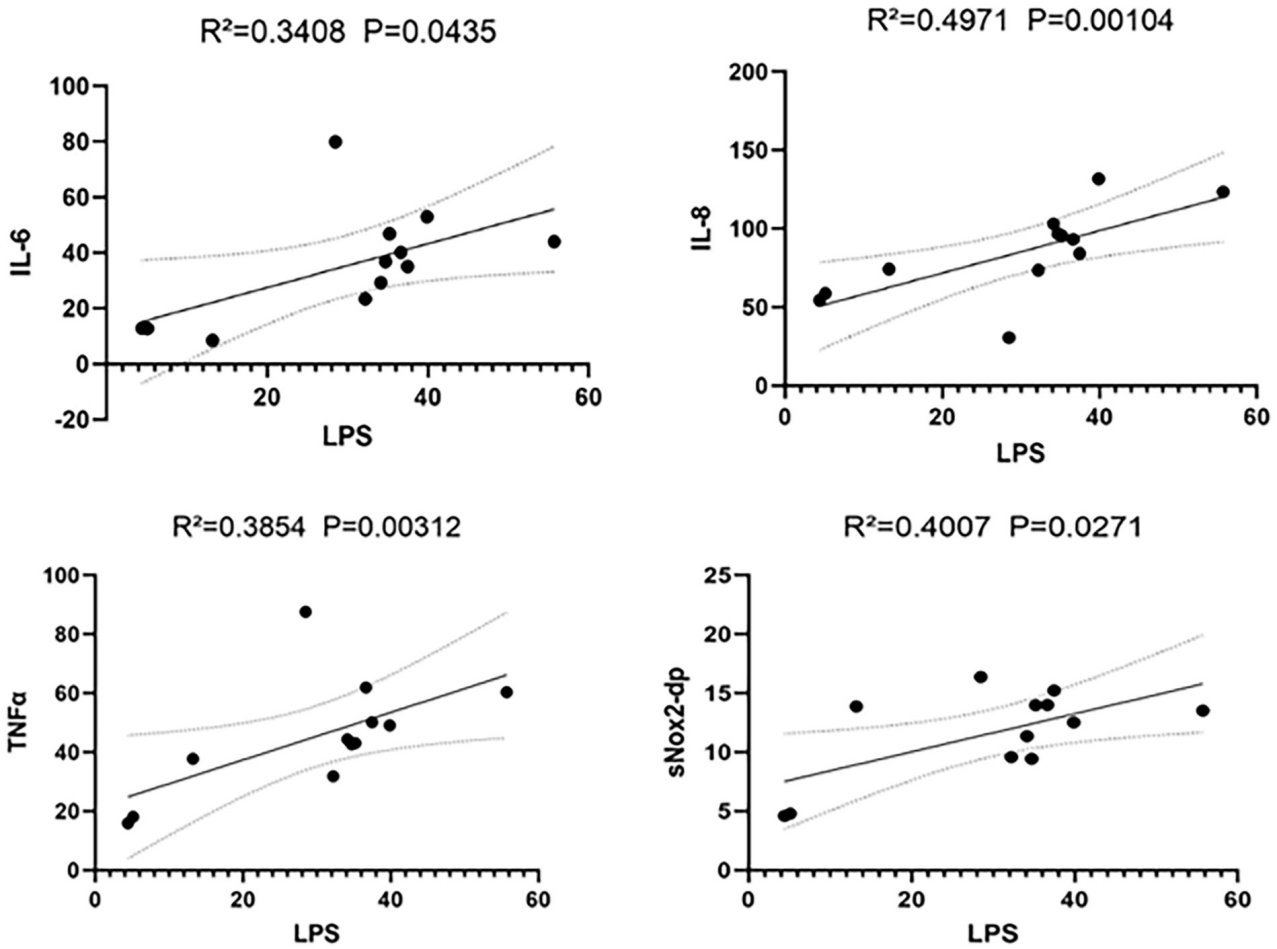
Correlation analysis between LPS and IL-6, IL-8, TNF-α, sNox2-dp in portal vein blood of cirrhotic patients.

The correlation analysis between LPS and other inflammatory markers in portal vein blood was also performed, positive correlations were observed between LPS and IL-6 (*r* = 0.3408, *P* = 0.044), IL-8 (*r* = 0.0.4971, *P* = 0.001), TNFα (*r* = 0.3854, *P* = 0.003), and sNox2-dp (*r* = 0.4007, *P* = 0.027) (Figure 3).

### Composition analysis of portal vein microflora between PVT and non-PVT groups

**Figure 4:**
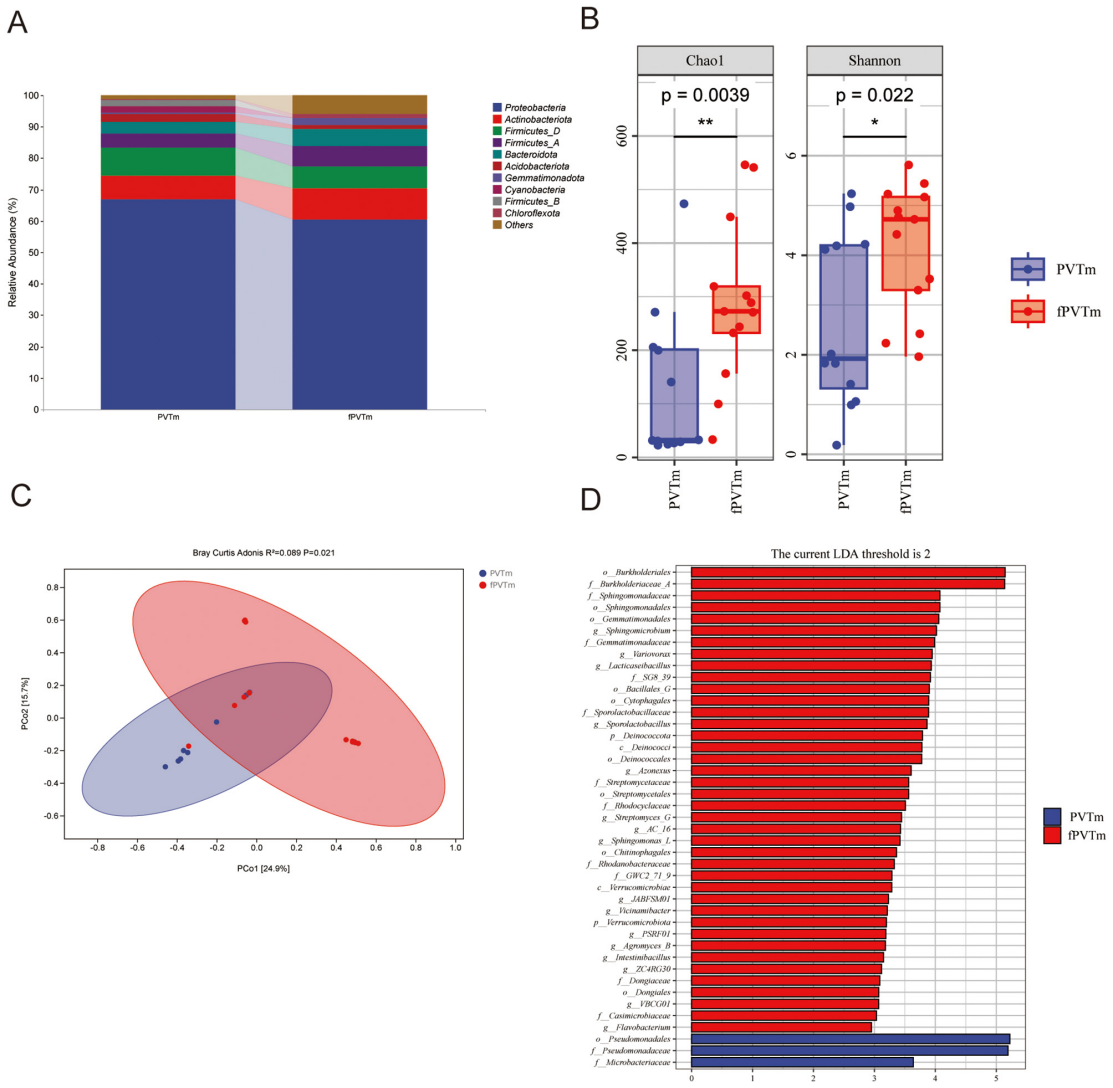
Microflora composition analysis of portal vein in PVT and non-PVT groups. **(A)** species community composition between two groups; **(B)** α-diversity analysis of the community composition in two groups; **(C)** β-diversity analysis of the community composition in two groups; **(D)** LEfSe analysis of the community composition differences in two groups. PVTm, portal vein blood of cirrhotic PVT patients; fPVTm, portal vein blood of cirrhotic non-PVT patients. The *p*-value is the value of the Kruskal-Wallis test. By default, it displays the significance markers of the Dunn's test *post-hoc* analysis, ^*^P < 0.05, ^**^P < 0.01.

Microflora composition analysis showed that four phyla were included in the portal vein blood microflora of cirrhotic PVT patients and non-cirrhotic PVT patients, among which the relative abundance of *Proteobacteria* in the portal vein blood of cirrhotic PVT patients was higher than that of cirrhotic non-PVT patients (67.03% vs. 60.45%), but the levels of *Firmicutes_D* (9.01% vs. 6.97%), *Actinobacteria* (7.26% vs. 10.05%), and *Firmicutes_A* (4.51% vs. 6.31%) in the portal vein blood of cirrhotic PVT patients were lower than those of cirrhotic non-PVT patients (Figure 4A). Chao1 (*P* = 0.004) and Shannon (*P* = 0.022) diversity indices showed significant differences of α-diversity in the portal blood of cirrhotic PVT patients and non-PVT patients (Figure 4B). The Bray-Curtis distance algorithm also revealed a significant difference (*P* < 0.05) of β-diversity in portal vein blood microflora of cirrhotic PVT patients and non-cirrhotic PVT patients (Figure 4C). With LDA score (log10) = 2 as the cutoff value to screen for important bacterial biomarkers, LEfSe analysis explored 43 species with significant differences in portal vein blood of cirrhotic PVT and non-PVT patients, among them, 2 species (*Pseudomonas, Microbacteriaceae*) significantly enriched in the portal vein blood microflora of cirrhotic PVT patients, while the remaining 40 species (*Burkholderiales, Burkholderiales, Sphingomonas, Sphingomonadales, Gemmatimonadales*, etc.) were significantly enriched in the portal vein microflora of cirrhotic non-PVT patients (Figure 4D).

### Composition analysis of PVT patients' microflora between portal and peripheral vein blood

**Figure 5:**
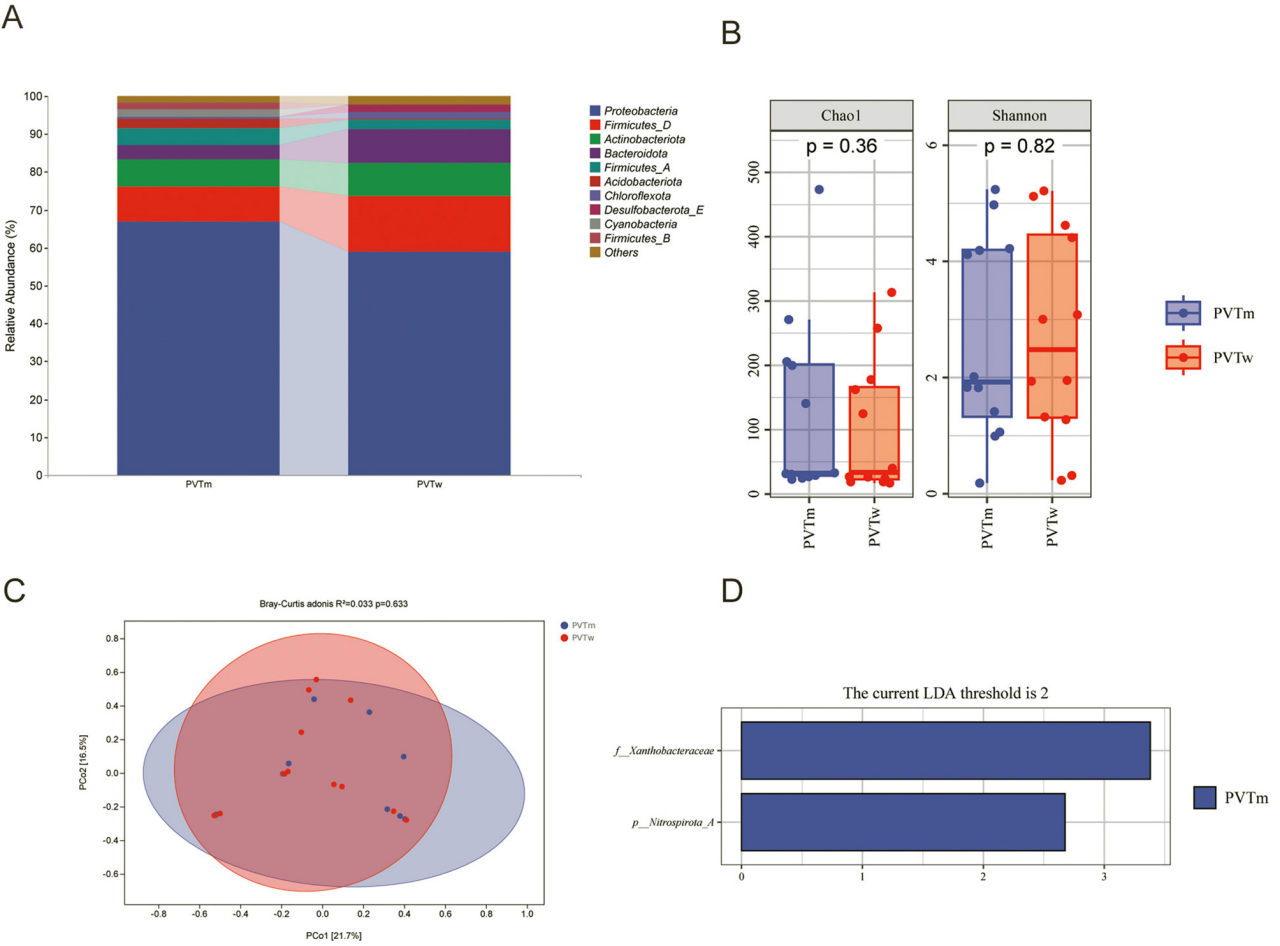
Analysis of portal and peripheral venous blood microflora in PVT group. **(A)** species community composition between two groups; **(B)** α-diversity analysis of the community composition in two groups; **(C)** β-diversity analysis of the community composition in two groups; **(D)** LEfSe analysis of the community composition differences in two groups. PVTm, portal vein blood of cirrhotic PVT patients; PVTw, peripheral venous blood of cirrhotic PVT patients.

Microflora composition analysis showed that the portal peripheral vein blood microflora in 12 cirrhotic PVT patients included four phyla, with the *Proteobacteria* being the most predominant, followed by the *Firmicutes, Actinomycetes*, and *Bacteroidetes*. The relative abundance in portal blood was higher than peripheral blood of *Proteobacteria* (67.03% vs. 59.05%), but lower than the peripheral blood of *Actinomycetes* (7.26% vs. 8.90%), *Bacteroidota* (3.79% vs. 8.85%), and *Firmicutes-D* (9.01% vs. 14.50%) (Figure 5A). Bacterial α-diversity showed that there was no significant difference between portal vein and peripheral blood of cirrhotic PVT patients (chao1: *P* = 0.36; Shannon: *P* = 0.82) (Figure 5B). Bray-Curtis distance algorithm showed that bacterial β-diversity was not significantly different between portal vein and peripheral blood of cirrhotic PVT patients (Figure 5C). With LDA score (log10) = 2 as the cutoff value to screen for important bacterial biomarkers, LEfSe analysis showed significant enrichment of *Nitrospirota* and *Xanthobacteraceae* in portal vein blood (Figure 5D).

## Discussion

The study revealed that the inflammatory marker concentrations in the portal vein blood of cirrhotic PVT patients were higher than those in peripheral blood, while the portal vein blood obtained from cirrhosis patients with PVT showed even higher concentrations of inflammatory markers compared to non-PVT patients. The microflora composition also differed significantly in portal vein blood obtained from PVT and non-PVT cirrhosis patients.

The LPS concentration increases with the severity of liver disease in cirrhotic patients (11, 12). Briefly, LPS could induce the production of a variety of diverse cytokines such as TNF-α, IL-1, IL-6, and other cytokines (13, 14). However, the effect of LPS in PVT is still controversial, Georgescu reported that a strong positive correlation was observed between PVT severity and TNF-alpha, their results also revealed that the underlying reason could be associated with gut microbiota (15), while Xu et al. (16) reported that TNF-α level in the PVT group was significantly lower than that in the non-PVT group. In this study, results showed there was a positive correlation between LPS and TNFα, IL-6, and IL-8, which was partially consistent with Georgescu's study, but the conflicts of previous results on LPS and PVT indicated that future studies should be performed to further understand the mechanism of LPS on PVT. Elevated portal vein pressure and altered hepatic metabolism in cirrhotic patients can disrupt the intestinal barrier, resulting in bacterial translocation (17, 18), and elevated concentrations of pro-inflammatory cytokines leading to the development of a localized highly inflammatory state (19). Our findings further corroborate this conclusion. In the current study, we observed that levels of inflammatory markers were significantly elevated in portal vein blood compared to those in peripheral vein blood.

In a recent study, Praktiknjo et al. (20) found that there was a concentration gradient between portal blood and peripheral blood for LPS, VWF, and factor VIII (FVIII) in decompensated cirrhotic patients treated with TIPS for ascites or variceal bleeding, which means LPS may promote endothelial injury and hypercoagulability. A study by Huang et al. (21) showed that IL-6, IL-8, TNFα, calcitoninogen, and C-reactive protein were significantly elevated in cirrhotic PVT patients with a history of esophageal and gastric variceal bleeding, which is consistent with the study results that increased LPS, IL-6, IL-8, and TNFα in the portal blood was found in patients with PVT, which may indicated that potential association between local endothelial injury, thrombosis formation and inflammatory activation.

As a marker of oxidative stress, NOX2 activity plays an important role in vascular disease because it can promote arterial vasoconstriction by inactivating nitric oxide, a potent vasodilator molecule, and thrombosis by promoting platelet aggregation (22). In the present study, soluble nox2-derived peptide sNOX2-dp was found to be significantly increased in the portal veins of cirrhotic PVT patients compared with those of peripheral veins, and its concentration was significantly correlated with the LPS concentration. The study showed that the concentrations of LPS, IL-6, TNFα, and sNox2-dp in the portal vein of cirrhotic PVT patients were significantly higher than those of cirrhotic non-PVT patients, without significant differences in peripheral blood concentrations, which further demonstrates that there is a greater accumulation of inflammatory factors in the local environment of the portal vein of cirrhotic PVT patients accompanied by oxidative stress.

Cirrhotic patients generally have a decrease in the diversity of intestinal microflora and a relative or absolute increase in the opportunistic microflora (23). However, the mechanism by how intestinal microflora affects PVT remains unclear. Chen et al. found that *Bacillota* and *Bacteroidota* are dominant in the fecal microorganism community of cirrhotic patients compared with healthy individuals, with an increased proportion of potentially pathogenic bacteria such as *Enterobacteriaceae* and *Streptococcaceae* as well as a decreased proportion of beneficial bacteria such as *Lachnospiraceae* (24). Robert et al. found that the domination of *Proteobacteria* in the microbial community of plasma and the correlation between systemic inflammation and blood microbiota in a study of 7 decompensated cirrhotic patients who underwent TIPS to extract portal vein, central vein, hepatic vein, and peripheral blood. It has been found that bacteria from the *Bacteroidota* and *Bacillota* are generally associated with health, with increased proportions of *Proteobacteria* being associated with inflammation and disease, and that *Proteobacteria* is generally found at a higher frequency in the human gut (25), which may be related to the fact that Gram-negative *Proteobacteria* produces a hexacylased lipopolysaccharide (LPS), which can promote intestinal inflammation (5). Compared to the healthy population, cirrhotic patients have a slower intestinal transit time, an overgrowth of intestinal bacteria, and an altered fecal microbial profile with enrichment of *Proteobacteria* and *Fusobacteria* as well as a decrease in *Bacteroidota* (26, 27), which is consistent with the previous studies (28). In the study, it was observed that the proportion of *Proteobacteria* was increased in portal vein blood than in peripheral vein blood, and the portal vein blood of PVT patients also showed a higher *Proteobacteria* proportion than the portal vein blood of non-PVT patients. In addition, the α-diversity abundance of the portal vein microflora was decreased in cirrhotic PVT patients compared with cirrhotic non-PVT patients, and the β-diversity in the microflora distribution differed significantly, suggesting that the distribution of the portal microflora of PVT patients was different from non-PVT patients, showing the potential diagnostic value in predicting the occurrence of cirrhotic PVT.

It was found that there was a correlation between portal vein blood microflora and inflammatory markers TNFα and snox2-dp at the genus level in cirrhotic PVT patients, with phenylobact erium significantly correlated with D-dimer on clinical parameters (29), which can be interpreted as the ability of these genera of bacteria to promote inflammation through the inflammatory vesicle cascade response, and the possible involvement of the bacteria in PVT. The lefse data of the present study demonstrated that *Pseudomonas* was significantly enriched in the portal vein microflora of cirrhotic PVT patients. In addition, the above species were positively correlated with each other in the network of cirrhotic microflora. Although these facts revealed associations between increased abundance of these microflora and PVT, the underlying mechanism should be further examined and validated in future prospective studies.

In future studies, we will continue to enroll cirrhotic patients who have undergone TIPS for further analysis and validation. We will also consider conducting a multi-center study.

## Limitation

However, this study still has several limitations. First, this is a single center study with a limited sample size; future large-scale prospective studies should be performed to validate the results of this study. Second, the correlation between inflammatory markers and microflora was not performed due to the large amount of microbiological species. The microflora composition also differed significantly across PVT and non-PVT patients. Thirdly, due to the limitation of collected blood samples, the microbial load analysis and absolute quantification experiments were not performed, the future analysis will try to collect more samples and perform more experiments. Finally, this study only revealed the possible correlation between blood flora change and PVT, the underlying mechanism was not studied and required future investigation.

## Conclusion

This study revealed that the concentration of inflammatory markers was higher in portal vein blood than in peripheral vein blood, while portal vein blood obtained from cirrhosis patients with PVT showed even higher concentrations of inflammatory markers compared to non-PVT patients. The microflora composition also differed significantly in portal vein blood obtained from PVT and non-PVT cirrhosis patients.

## Data Availability

The original contributions presented in the study are included in the article/Supplementary material, further inquiries can be directed to the corresponding author.

## Supplementary material

Supplementary Figure S1 Inflammatory markers in portal and peripheral venous blood of cirrhotic patients.

Supplementary Figure S2 Inflammatory markers in portal and peripheral venous blood of PVT group.
