# Supplementary figures and images for: Comparison of inflammatory markers and microflora in cirrhotic patients with portal vein thrombosis: a retrospective study
Source: Front Med (Lausanne). 2026 Jan 12;12:1680970. doi: 10.3389/fmed.2025.1680970 (PMC12832706; doi:10.3389/fmed.2025.1680970)

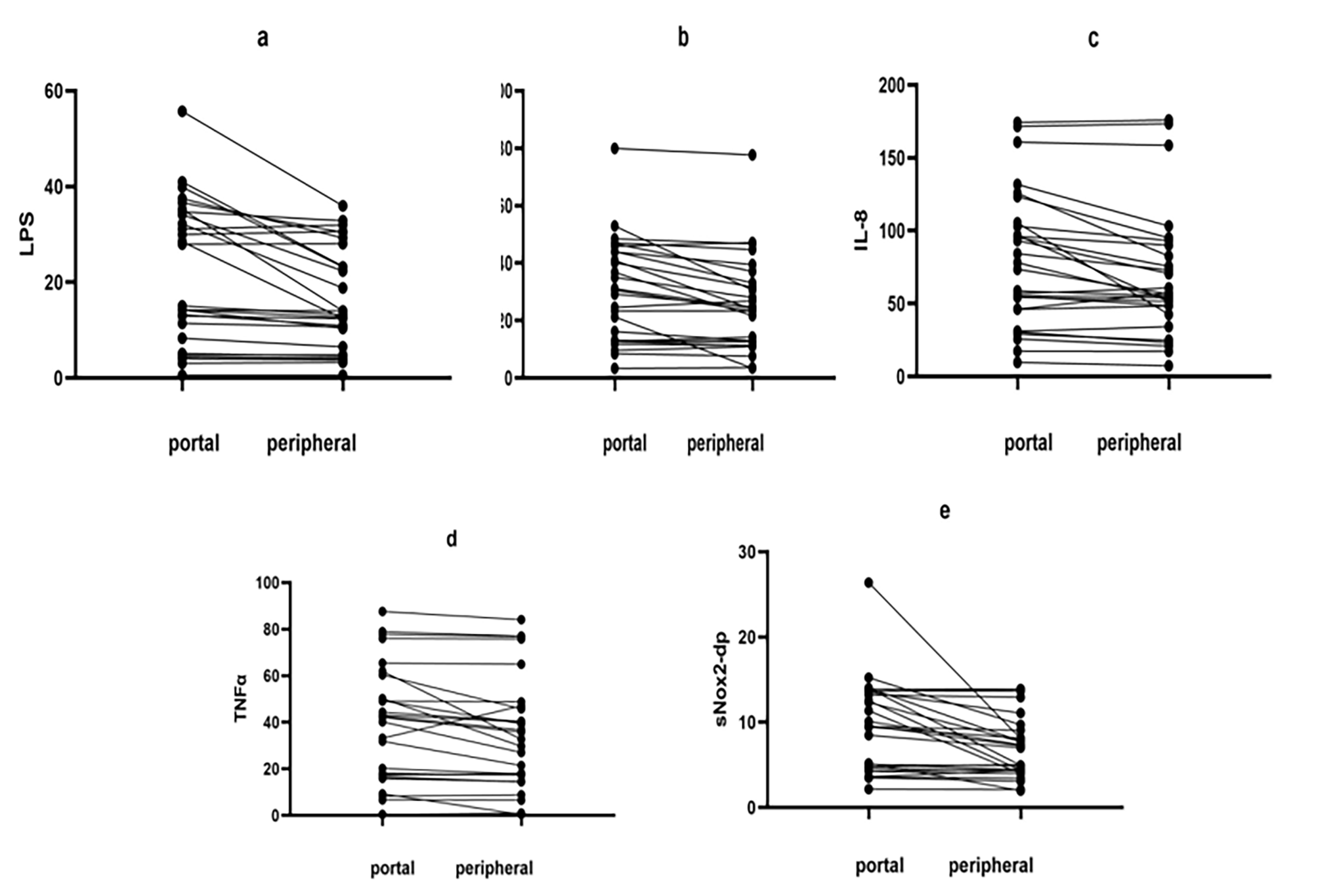

Supplement: Supplementary Figure S1 — Inflammatory markers in portal and peripheral venous blood of cirrhotic patients. [file Image_1.tif]

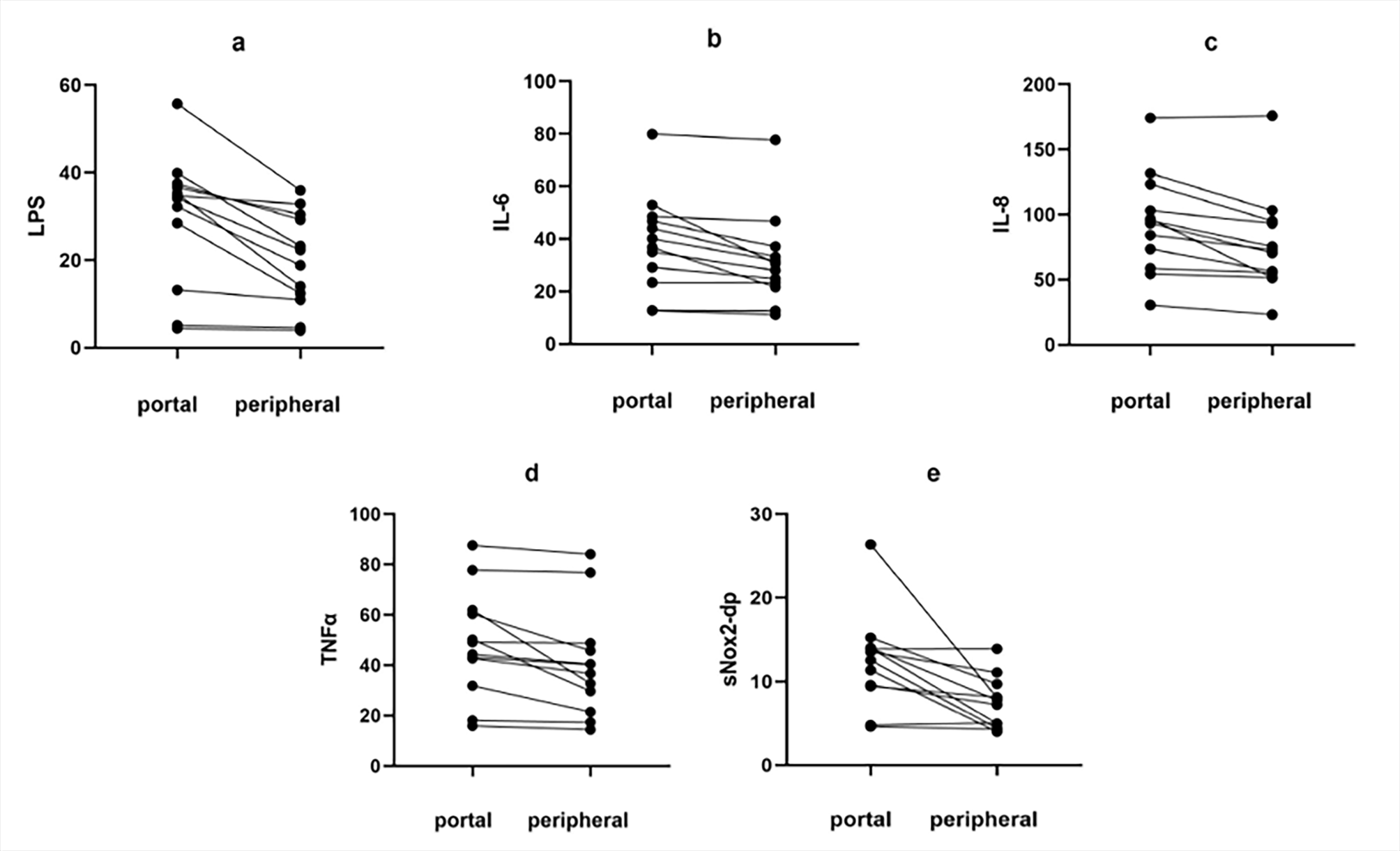

Supplement: Supplementary Figure S2 — Inflammatory markers in portal and peripheral venous blood of PVT group. [file Image_2.tif]
